# Supplementary figures and images for: SARS-CoV-2 seroprevalence in three Kenyan health and demographic surveillance sites, December 2020-May 2021
Source: PLOS Glob Public Health. 2022 Aug 18;2(8):e0000883. doi: 10.1371/journal.pgph.0000883 (PMC10021917; doi:10.1371/journal.pgph.0000883)

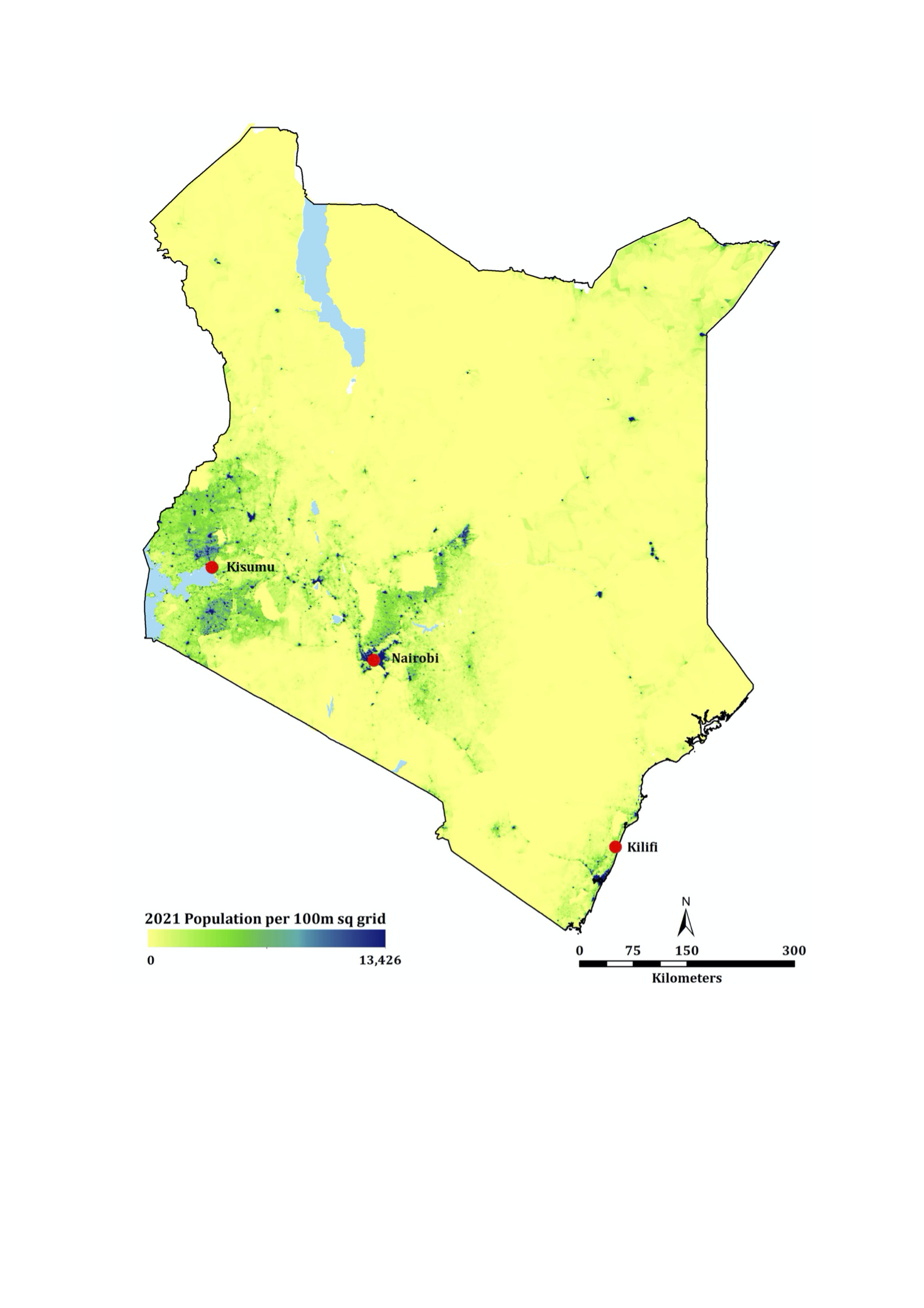

Supplement: S1 Fig — We used the Kenya boundaries outline shapefile from the Humanitarian data exchange platform https://data.humdata.org/dataset/cod-ab-ken. Population data were downloaded in raster format from WorldPop at 1km spatial resolution https://www.worldpop.org/. (TIFF) [file pgph.0000883.s001.tiff]

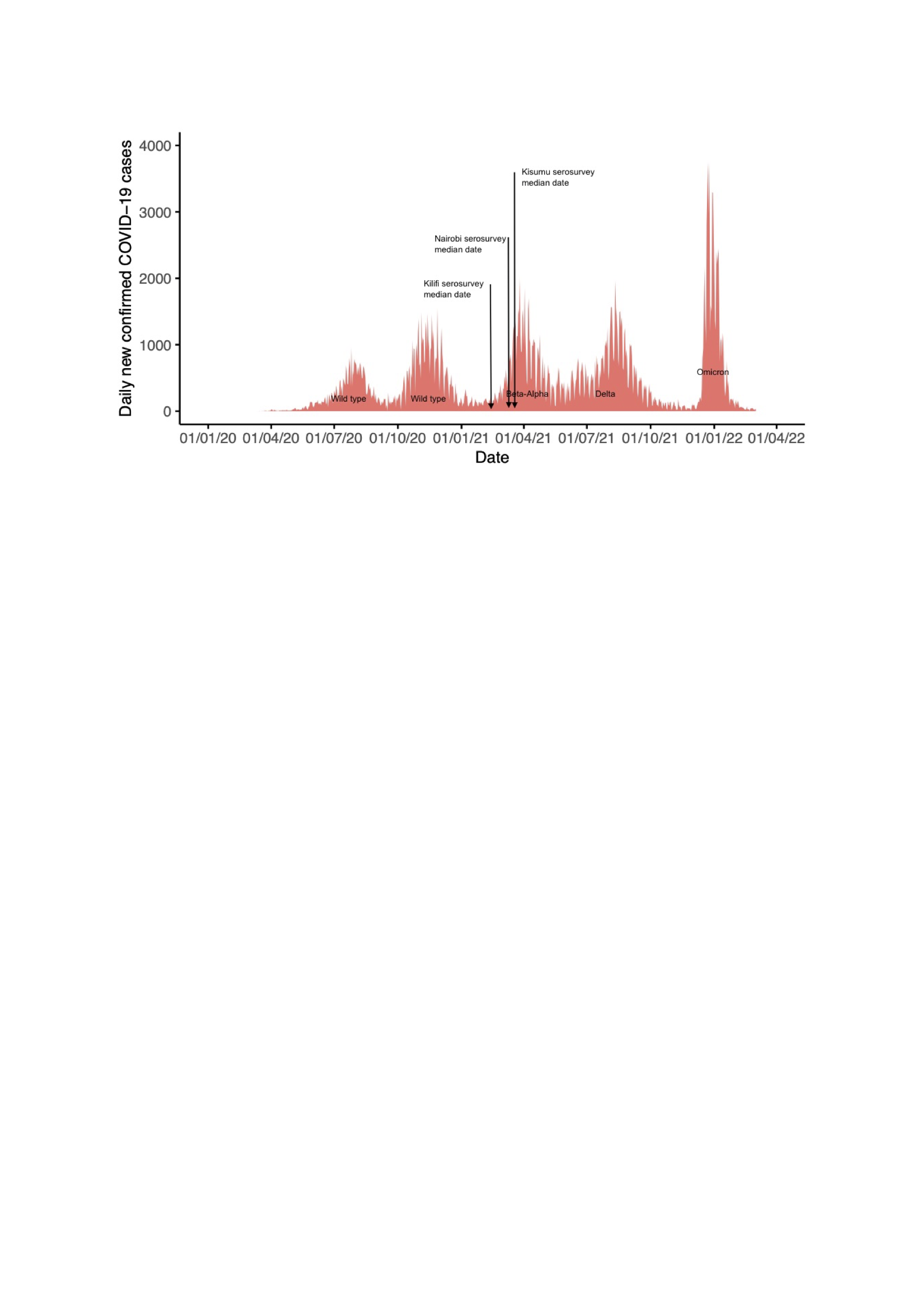

Supplement: S2 Fig — The orange data series are the daily number of new cases of COVID-19 in Kenya. The predominant variant behind each wave is denoted at the base of each wave. Data source: COVID-19 cases were obtained from Our World in Data (https://ourworldindata.org). (TIFF) [file pgph.0000883.s002.tiff]

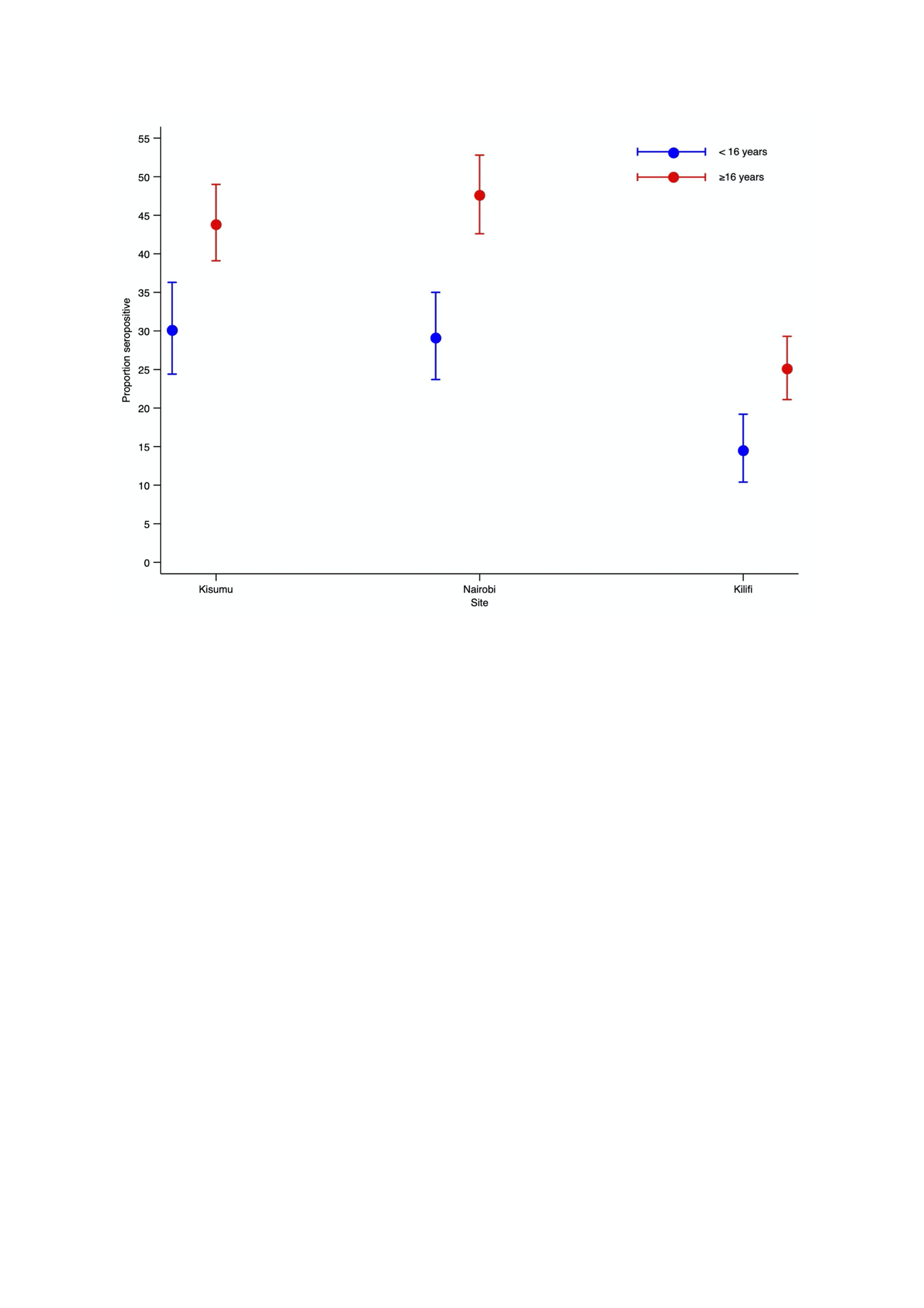

Supplement: S3 Fig — (TIFF) [file pgph.0000883.s003.tiff]
