# Supplementary material for: SARS-CoV-2 seroprevalence in three Kenyan health and demographic surveillance sites, December 2020-May 2021
Source: PLOS Glob Public Health. 2022 Aug 18;2(8):e0000883. doi: 10.1371/journal.pgph.0000883 (PMC10021917; doi:10.1371/journal.pgph.0000883)
Supplement: S1 Table — § Figures represent percentages with 95% credible intervals in parentheses. Bayesian threshold analysis adjusted for test performance and underlying population structure using multilevel regression and poststratification (DOCX) [file pgph.0000883.s004.docx]

S1 Table: Seroprevalence by HDSS site and study period

|  |  | **HDSS site** | | | | | | | | | | | | | |
| --- | --- | --- | --- | --- | --- | --- | --- | --- | --- | --- | --- | --- | --- | --- | --- |
|  |  | Kisumu | | | |  | Nairobi | | | |  | Kilifi | | | |
|  |  |  | Sero-positive | Bayesian  adjustment§ | |  |  | Sero-positive | Bayesian  adjustment§ | |  |  | Sero-positive | Bayesian  adjustment§ | |
| **Period** |  | N | n | % | 95% CI |  | N | n | % | 95% CI |  | N | n | % | 95% CI |
| 01 Dec 20- 31 Dec 20 |  | - | - | - |  |  | - | - | - |  |  | 162 | 27 | 14.5 | 9.1-21 |
| 01 Jan 21- 31 Jan 21 |  | - | - | - |  |  | 124 | 39 | 32.4 | 23.1-42.4 |  | 113 | 23 | 20.9 | 13.4-29.8 |
| 01 Feb 21-28 Feb 21 |  | 214 | 69 | 36.0 | 28.2-44.4 |  | 144 | 48 | 35.9 | 27.3-45.2 |  | 239 | 41 | 17.7 | 12.5-23.5 |
| 01 Mar 21-31 Mar 21 |  | 218 | 81 | 39.9 | 32.3-47.8 |  | 186 | 77 | 47.1 | 38.0-56.6 |  | 197 | 47 | 24.6 | 17.9-31.7 |
| 01 Apr 21-30 Apr 21 |  | 204 | 73 | 36.6 | 28.9-44.5 |  | 283 | 104 | 38.0 | 31.1-45.3 |  | 145 | 35 | 24.7 | 17.5-32.6 |
| 01 May 21-31 May 21 |  | 217 | 85 | 42.0 | 34.7-50.0 |  | 113 | 54 | 50.2 | 39.7-61.1 |  | - | - | - |  |
| **01 Dec 20-31 May 21** |  | **853** | **308** | **38.1** | **34.1-42.4** |  | **850** | **322** | **40.4** | **36.0-45.0** |  | **856** | **173** | **19.8** | **16.8-23.1** |

^§^ Figures represent percentages with 95% credible intervals in parentheses. Bayesian threshold analysis adjusted for test performance and underlying population structure using multilevel regression and poststratification
