# Supplementary material for: SARS-CoV-2 seroprevalence in three Kenyan health and demographic surveillance sites, December 2020-May 2021
Source: PLOS Glob Public Health. 2022 Aug 18;2(8):e0000883. doi: 10.1371/journal.pgph.0000883 (PMC10021917; doi:10.1371/journal.pgph.0000883)
Supplement: S2 Table — ± Performance adjusted Bayesian threshold analysis adjusted for test performance and underlying population structure using multilevel regression and poststratification. Figures represent percentages with 95% credible intervals in parentheses. § Kisumu data were collected from 01 Feb 2021–27 May 2021. (DOCX) [file pgph.0000883.s005.docx]

S2 Table: Age specific seroprevalence at three HDSS sites in Kenya

|  | **Study Period** | | | | | | | | | | | | | | | | | |
| --- | --- | --- | --- | --- | --- | --- | --- | --- | --- | --- | --- | --- | --- | --- | --- | --- | --- | --- |
|  | **01 Dec 2020-29 Jan 2021** | | | |  | **30 Jan 2021-29 Mar2021** | | | | |  | **30 Mar 2021-27 May2021** | | |  | **Overall** | | |
|  | **N** | | **+VE** | **Sero-prevalence**^±^ |  | **N** | | **+VE** | | **Sero-prevalence**^±^ |  | **N** | **+VE** | **Sero-prevalence**^±^ |  | **N** | **+VE** | **Sero-prevalence**^±^ |
| *Kisumu^§^* |  |  | | | | | | | | | | | | | | | | |
| <16 years | - | | - | - |  | | 165 | 40 | 28.9 (21.3-38) | |  | 144 | 42 | 34.4 (26.1-42.4) |  | 309 | 82 | 30.1 (24.4-36.3) |
| 16-24 years | - | | - | - |  | | 44 | 20 | 44.9 (32.9-58.9) | |  | 46 | 20 | 43.7 (33-56.1) |  | 90 | 40 | 45.2 (36.1-55.4) |
| 25-34 years | - | | - | - |  | | 37 | 18 | 46.4 (33.7-61.9) | |  | 66 | 20 | 36.4 (25.9-46.3) |  | 103 | 38 | 39.7 (31.2-48.6) |
| 35-44 years | - | | - | - |  | | 53 | 24 | 45.2 (33.9-58.5) | |  | 47 | 24 | 46.4 (35.2-61) |  | 100 | 48 | 47.4 (38.1-57.7) |
| 45-54 years | - | | - | - |  | | 41 | 15 | 40.0 (27.9-52.9) | |  | 61 | 27 | 43.6 (33.5-55.6) |  | 102 | 42 | 42.6 (33.9-52.0) |
| 55-64 years | - | | - | - |  | | 48 | 18 | 40.2 (28.6-52.9) | |  | 51 | 20 | 40.8 (30.6-52.4) |  | 99 | 38 | 40.8 (32.1-50.1) |
| ≥ 65 years | - | | - | - |  | | 29 | 11 | 40.5 (27.3-55) | |  | 21 | 9 | 42.7 (29.9-58.2) |  | 50 | 20 | 42.0 (31.1-54.1) |
| *Nairobi* |  |  | | | | | | | | | | | | | | | | |
| <16 years | 33 | | 8 | 28.4 (15.6-42.3) |  | 130 | | 30 | | 26.5 (18.7-35.4) |  | 154 | 45 | 33.7 (25.6-42.2) |  | 317 | 83 | 29.1 (23.7-35.0) |
| 16-24 years | 8 | | 1 | 27.9 (9.8-47.5) |  | 41 | | 25 | | 59.5 (44-74.9) |  | 40 | 14 | 41.2 (27.9-54.8) |  | 89 | 40 | 47.4 (37.4-58.0) |
| 25-34 years | 14 | | 2 | 26.1 (9.6-43.4) |  | 26 | | 8 | | 36.7 (20.9-54) |  | 52 | 23 | 47.5 (35.4-60.6) |  | 92 | 33 | 39.8 (30.0-49.9) |
| 35-44 years | 6 | | 2 | 32.1 (13.5-56.7) |  | 38 | | 21 | | 54.6 (39.5-70.6) |  | 57 | 28 | 51.1 (38.7-64.2) |  | 101 | 51 | 52.2 (42.1-62.6) |
| 45-54 years | 8 | | 3 | 32.7 (14.9-56.9) |  | 44 | | 20 | | 46.9 (33.1-61.7) |  | 47 | 20 | 46.4 (33.7-59.6) |  | 99 | 43 | 46.2 (36.5-56.3) |
| 55-64 years | 14 | | 5 | 33.0 (16.6-54) |  | 44 | | 12 | | 32.4 (19.7-46.1) |  | 43 | 26 | 58.2 (42.9-73.5) |  | 101 | 43 | 45.4 (35.8-55.3) |
| ≥ 65 years | 12 | | 8 | 43.8 (23.4-72.5) |  | 25 | | 12 | | 48.4 (31.5-66.6) |  | 14 | 9 | 55.3 (37.8-76.5) |  | 51 | 29 | 55.5 (42.6-69.3) |
| *Kilifi* |  |  | | | | | | | | | | | | | | | | |
| <16 years | 93 | | 6 | 9.8 (4-17.3) |  | 142 | | 17 | | 15.7 (9.5-22.2) |  | 74 | 17 | 25.0 (15.8-35.1) |  | 309 | 40 | 14.5 (10.4-19.2) |
| 16-24 years | 29 | | 5 | 18.9 (8.5-32.3) |  | 43 | | 12 | | 24.8 (16.1-37.1) |  | 21 | 3 | 21.8 (8.4-36.5) |  | 93 | 20 | 22.5 (15.1-31.0) |
| 25-34 years | 28 | | 5 | 19.3 (8.8-33) |  | 53 | | 13 | | 23.5 (15.4-34.1) |  | 20 | 3 | 22.2 (8.6-37.2) |  | 101 | 21 | 22.0 (15.0-30.0) |
| 35-44 years | 31 | | 7 | 22.3 (11.2-37) |  | 49 | | 14 | | 25.4 (16.6-37.8) |  | 21 | 12 | 43.5 (23.9-67.7) |  | 101 | 33 | 31.4 (22.6-41.6) |
| 45-54 years | 28 | | 10 | 30.5 (16.5-48.4) |  | 59 | | 11 | | 20.5 (12.9-29.3) |  | 13 | 4 | 29.3 (13.9-49.7) |  | 100 | 25 | 25.2 (17.7-33.8) |
| 55-64 years | 41 | | 7 | 18.5 (9.1-30.3) |  | 51 | | 10 | | 21.0 (12.9-30.4) |  | 9 | 2 | 26.4 (10.2-47) |  | 101 | 19 | 20.5 (13.6-28.1) |
| ≥ 65 years | 22 | | 8 | 29.8 (15.2-49.2) |  | 24 | | 6 | | 23.0 (13.5-36.1) |  | 5 | 1 | 26.3 (8.8-48.7) |  | 51 | 15 | 27.3 (17.8-39.4) |
