# Supplementary material for: SARS-CoV-2 seroprevalence in three Kenyan health and demographic surveillance sites, December 2020-May 2021
Source: PLOS Glob Public Health. 2022 Aug 18;2(8):e0000883. doi: 10.1371/journal.pgph.0000883 (PMC10021917; doi:10.1371/journal.pgph.0000883)
Supplement: S3 Table — ± Kilifi HDSS population as at 18 Feb 2021 was used as the standard population. Prevalence estimates were adjusted for performance characteristics of assay used as described in methods section. § Figures represent percentages with 95% credible intervals in parentheses. Bayesian threshold analysis adjusted for test performance and underlying population structure using multilevel regression and poststratification. (DOCX) [file pgph.0000883.s006.docx]

S3 Table: Sex-stratified seroprevalence by HDSS site and study period

|  |  |  |  | **HDSS Site** | | | | | | | | | | | | | | | | |
| --- | --- | --- | --- | --- | --- | --- | --- | --- | --- | --- | --- | --- | --- | --- | --- | --- | --- | --- | --- | --- |
|  |  |  |  | Kisumu | | | | |  | Nairobi | | | | | |  | Kilifi | | | |
| **Period** |  | **Sex** |  | N | Sero +VE | | Classical adjustment^±^ | Bayesian adjustment^§^ |  | N | Sero +VE | Classical adjustment^±^ | | | Bayesian adjustment^§^ |  | N | Sero +VE | Classical adjustment^±^ | Bayesian adjustment^§^ |
| 01 Dec 20- 31 Dec 20 |  | Female |  | - | - | - | | - |  | - | - | - | - | | |  | 96 | 18 | 11.2 (7.7-14.6) | 17.0 (9.8-25.7) |
|  |  | Male |  | - | - | - | | - |  | - | - | - | - | | |  | 66 | 9 | 13.1 (4.1-22.1) | 11.6 (4.7-20.7) |
| 01 Jan 21- 31 Jan 21 |  | Female |  | - | - | - | | - |  | 74 | 23 | 16.1 (10.3-21.9) | | 31.9 (20.6-44.2) | |  | 61 | 10 | 14.3 (9.9-18.6) | 16.4 (7.5-27.6) |
|  |  | Male |  | - | - | - | | - |  | 50 | 16 | 27.0 (23.7-30.4) | | 32.9 (19.6-48) | |  | 52 | 13 | 17.6 (12.1-23.1) | 25.8 (14.3-39.4) |
| 01 Feb 21-28 Feb 21 |  | Female |  | 117 | 37 | 39.5 (32.6-46.4) | | 34.8 (25.3-45.1) |  | 64 | 21 | 36.6 (33.8-39.5) | | 35.2 (23-48.6) | |  | 140 | 21 | 16.0 (12.3-19.7) | 15.1 (9.1-22.2) |
|  |  | Male |  | 97 | 32 | 34.0 (28.3-39.8) | | 37.4 (26.4-49.3) |  | 80 | 27 | 25.2 (20.7-29.8) | | 36.5 (25.2-48.8) | |  | 99 | 20 | 21.0 (16.3-25.6) | 20.7 (12.6-29.7) |
| 01 Mar 21-31 Mar 21 |  | Female |  | 123 | 47 | 35.9 (29.6-42.3) | | 40.9 (30.8-51.1) |  | 78 | 38 | 57.2 (50.5-63.9) | | 52.6 (39.9-65.5) | |  | 96 | 27 | 26.3 (22.7-29.9) | 28.6 (18.7-39.2) |
|  |  | Male |  | 95 | 34 | 32.2 (24.9-39.4) | | 38.8 (27.9-50.2) |  | 108 | 39 | 45.2 (40.3-50.0) | | 42.2 (31.2-54.0) | |  | 101 | 20 | 18.5 (12.6-24.4) | 20.2 (12.6-29.3) |
| 01 Apr 21-30 Apr 21 |  | Female |  | 106 | 43 | 37.2 (30.9-43.6) | | 41.6 (31.2-52.5) |  | 128 | 41 | 23.6 (19.0-28.2) | | 33.0 (24.2-42.6) | |  | 66 | 16 | 21.3 (16.3-26.3) | 23.7 (13.7-35.6) |
|  |  | Male |  | 98 | 30 | 38.9 (35.1-42.8) | | 31.2 (21.5-41.9) |  | 155 | 63 | 42.3 (36.3-48.3) | | 42.4 (33.2-52.2) | |  | 79 | 19 | 29.5 (23.4-35.6) | 25.7 (16.0-36.6) |
| 01 May 21-31 May 21 |  | Female |  | 114 | 48 | 45.1 (37.1-53.0) | | 45.4 (35.4-55.8) |  | 56 | 30 | 44.3 (38.5-50.2) | | 56.6 (41.6-71.5) | |  | - | - | - | - |
|  |  | Male |  | 103 | 37 | 45.3 (39.8-50.8) | | 38.3 (28.2-49.3) |  | 57 | 24 | 49.7 (44.6-54.9) | | 44.7 (30.9-59.4) | |  | - | - | - | - |

^±^ Kilifi HDSS population as at 18 Feb 2021 was used as the standard population. Prevalence estimates were adjusted for performance characteristics of assay used as described in methods section.

^§^ Figures represent percentages with 95% credible intervals in parentheses. Bayesian threshold analysis adjusted for test performance and underlying population structure using multilevel regression and poststratification
