# Supplementary material for: SARS-CoV-2 seroprevalence in three Kenyan health and demographic surveillance sites, December 2020-May 2021
Source: PLOS Glob Public Health. 2022 Aug 18;2(8):e0000883. doi: 10.1371/journal.pgph.0000883 (PMC10021917; doi:10.1371/journal.pgph.0000883)
Supplement: S4 Table — (DOCX) [file pgph.0000883.s007.docx]

S4 Table: Age and Sex-stratified seroprevalence by HDSS site for entire study period

|  | **Entire study period** | | | |
| --- | --- | --- | --- | --- |
|  | Sample size | positive | classical test-adjusted and population adjusted seroprevalence | Bayesian population-weighted, test-adjusted seroprevalence |
| **Kisumu** |  |  |  |  |
| Overall | 853 | 308 | 39.0 (34.8-43.1) | 38.1 (34.1-42.4) |
|  |  |  |  |  |
| **Sex** |  |  |  |  |
| Female | 460 | 175 | 40.0 (35.0-45.1) | 40.1 (34.8-45.5) |
| Male | 393 | 133 | 36.5 (31.2-41.8) | 36.1 (30.7-41.8) |
|  |  |  |  |  |
| **Age category (years)** |  |  |  |  |
| 0-9 | 199 | 43 | 25.6 (18.3-32.9) | 25.5 (19-32.8) |
| 10-19 | 150 | 53 | 37.4 (29.1-45.7) | 38.3 (30.6-46.3) |
| 20-29 | 102 | 42 | 44.4 (34.2-54.5) | 42.9 (33.9-52.5) |
| 30-39 | 101 | 46 | 48.9 (37.3-60.5) | 45.9 (36.5-55.9) |
| 40-49 | 100 | 38 | 42.7 (31.2-54.1) | 39.7 (30.8-49.3) |
| 50-59 | 101 | 46 | 50.0 (39.9-60.1) | 46.1 (36.7-56.3) |
| 60-69 | 79 | 33 | 42.1 (30.8-53.4) | 43.2 (33.3-54) |
| 70-79 | 15 | 5 | 30.8 (16.2-45.4) | 38.5 (23.5-54.9) |
| 80+ | 6 | 2 | 25.3 (25.3-25.3) | 39.9 (22.6-58.7) |
|  |  |  |  |  |
| <15 (children) | 299 | 79 | 29.8 (23.9-35.7) | 28.6 (23.1-34.6) |
| 15+ (adults) | 554 | 229 | 44.2 (38.7-49.8) | 43.6 (38.8-48.6) |
| **Nairobi** |  |  |  |  |
| Overall | 850 | 322 | 39.6 (35.2-44.0) | 40.4 (36.0-45.0) |
|  |  |  |  |  |
| **Sex** |  |  |  |  |
| Female | 400 | 153 | 37.3 (31.7-42.9) | 40.0 (34.4-45.9) |
| Male | 450 | 169 | 41.3 (35.9-46.7) | 40.7 (35.1-46.5) |
| **Age category (years)** |  |  |  |  |
| 0-9 | 198 | 35 | 23.7 (15.9-31.4) | 20.6 (14.7-27.3) |
| 10-19 | 154 | 70 | 50.6 (41.0-60.2) | 48.3 (39.8-57.2) |
| 20-29 | 100 | 32 | 34.0 (23.5-44.6) | 35.5 (26.2-45.5) |
| 30-39 | 98 | 45 | 45.4 (35.2-55.6) | 48.5 (38.5-58.9) |
| 40-49 | 98 | 48 | 54.1 (42.9-65.4) | 51.4 (41.1-61.8) |
| 50-59 | 101 | 45 | 45.9 (35.4-56.5) | 47.3 (37.5-57.7) |
| 60-69 | 75 | 32 | 43.8 (30.6-57.0) | 45.4 (34.3-57.2) |
| 70-79 | 20 | 10 | 38.5 (21.1-55.8) | 49.7 (32.1-68.6) |
| 80+ | 6 | 5 | 82.5 (82.5-82.5) | 57.1 (33.9-83) |
|  |  |  |  |  |
| <15 (children) | 299 | 73 | 28.5 (22.3-34.7) | 28.3 (22.9-33.9) |
| 15+ (adults) | 551 | 249 | 44.0 (38.4-49.6) | 47.6 (42.6-52.8) |
| **Kilifi** |  |  |  |  |
| Overall | 856 | 173 | 20.0 (17.0-23.0) | 19.8 (16.8-23.1) |
|  |  |  |  |  |
| **Sex** |  |  |  |  |
| Female | 459 | 92 | 19.4 (15.3-23.5) | 19.2 (15.3-23.4) |
| Male | 397 | 81 | 20.8 (16.6-24.9) | 20.5 (16.3-25.0) |
|  |  |  |  |  |
| **Age category (years)** |  |  |  |  |
| 0-9 | 197 | 16 | 8.4 (3.9-12.9) | 10.2 (5.8-15.4) |
| 10-19 | 153 | 31 | 21.9 (14.7-29.1) | 21.3 (15.1-28.4) |
| 20-29 | 101 | 22 | 23.2 (14.9-31.6) | 22.6 (15.1-31.3) |
| 30-39 | 102 | 31 | 32.0 (22.4-41.6) | 30.3 (21.8-39.8) |
| 40-49 | 101 | 27 | 28.5 (19.4-37.6) | 27.1 (18.9-36.4) |
| 50-59 | 101 | 22 | 24.1 (14.8-33.5) | 22.6 (15.2-31.1) |
| 60-69 | 66 | 11 | 14.3 (6.7-22.0) | 18.8 (10.6-28.3) |
| 70-79 | 27 | 10 | 41.1 (28.1-54.1) | 31.4 (18.2-48.3) |
| 80+ | 8 | 3 | 34.1 (0-70.8) | 27.6 (12.6-50.2) |
|  |  |  |  |  |
| <15 (children) | 295 | 35 | 12.3 (8.2-16.5) | 12.5 (8.6-17.1) |
| 15+ (adults) | 561 | 138 | 26.0 (21.8-30.2) | 25.5 (21.5-29.7) |
|  |  |  |  |  |
