# Supplementary material for: SARS-CoV-2 seroprevalence in three Kenyan health and demographic surveillance sites, December 2020-May 2021
Source: PLOS Glob Public Health. 2022 Aug 18;2(8):e0000883. doi: 10.1371/journal.pgph.0000883 (PMC10021917; doi:10.1371/journal.pgph.0000883)
Supplement: S5 Table — § Per 5-year increase in age. ± Symptoms included the following: Abdominal pain, chest pain, cough, diarrhoea, fever and chills, headache, irritability and confusion, joint pains, loss of smell/taste, muscular pain, nausea and vomiting, runny nose, shortness of breath, sore throat and weakness. (DOCX) [file pgph.0000883.s008.docx]

S5 Table: Multivariable analysis of factors associated with presence of antibodies to SARS-CoV-2 in Kisumu, Nairobi, and Kilifi

| Characteristic |  | OR | | *(95% CI)* |  |  |
| --- | --- | --- | --- | --- | --- | --- |
| Sex |  |  |  | |  |  |
| Female |  | 1.0 | *–* | |  |  |
| Male |  | 0.92 | *(0.77-1.09)* | |  |  |
| Age category^§^ |  | 1.07 | *(1.05-1.09)* | |  |  |
| History of symptoms^±^ |  | 0.95 | *(0.79-1.16)* | |  |  |
| Location |  |  |  | |  |  |
| Kisumu |  | 1.0 | *–* | |  |  |
| Nairobi |  | 1.21 | *(0.97-1.50)* | |  |  |
| Kilifi |  | 0.54 | *(0.42-0.69)* | |  |  |
| Study period |  | 1.07 | *(1.00-1.14)* | |  |  |
| Education level |  |  |  | |  |  |
| None |  | 1.0 | *–* | |  |  |
| Primary |  | 1.36 | *(1.07-1.73)* | |  |  |
| Secondary |  | 1.65 | *(1.25-2.18)* | |  |  |
| Tertiary |  | 1.57 | *(1.04-2.38)* | |  |  |

^§^ Per 5-year increase in age

^±^ Symptoms included the following: abdominal pain, chest pain, cough, diarrhoea, fever and chills, headache, irritability and confusion, joint pains, loss of smell/taste, muscular pain, nausea and vomiting, runny nose, shortness of breath, sore throat and weakness
