# Supplementary material for: SARS-CoV-2 seroprevalence in three Kenyan health and demographic surveillance sites, December 2020-May 2021
Source: PLOS Glob Public Health. 2022 Aug 18;2(8):e0000883. doi: 10.1371/journal.pgph.0000883 (PMC10021917; doi:10.1371/journal.pgph.0000883)
Supplement: S1 Text — (DOCX) [file pgph.0000883.s009.docx]

**S1 Text: Stan code for Bayesian adjustment of prevalence estimates to account for test performance and population structure**

data {

int N_se; // denominator sensitivity

int N_sp; // denominator specificity

int x; // numerator sensitivity

int z; // numerator specificity

int y[7, 2]; // no. seropositives

int n[7, 2]; // no. samples

real pw[7, 2]; // proportion of population in each demographic subgroup

real tot_pw_age[7]; // proportion of population in each age group

real tot_pw_sex[2]; // proportion female and male

}

parameters {

real<lower=0,upper=1> se;

real<lower=0,upper=1> sp;

real bsex[2];

real bage[7];

real<lower=0> sd_age;

}

transformed parameters {

real<lower=0,upper=1> p[7, 2];

real<lower=0,upper=1> p_obs[7, 2];

for(a in 1:7){

for(s in 1:2){

p[a, s] = inv_logit(bage[a] +

bsex[s]);

p_obs[a, s] = se * p[a, s] +

(1 - sp) * (1 - p[a, s]);

}

}

}

model {

//priors

se ~ beta(1, 1);

sp ~ beta(1, 1);

bsex ~ normal(0, 10);

bage ~ normal(0, sd_age);

sd_age ~ normal(0, 0.5);

//likelihood

for(a in 1:7){

for(s in 1:2){

y[a, s] ~ binomial(n[a, s], p_obs[a, s]);

}

}

x ~ binomial(N_se, se);

z ~ binomial(N_sp, sp);

}

generated quantities {

real p_national = 0;

vector[7] p_age = rep_vector(0, 7);

vector[2] p_sex = rep_vector(0, 2);

for(a in 1:7){

for(s in 1:2){

p_national += p[a, s] * pw[a, s];

}

}

for(a in 1:7){

for(s in 1:2){

p_age[a] += p[a, s] * pw[a, s]/tot_pw_age[a];

}

}

for(s in 1:2){

for(a in 1:7){

p_sex[s] += p[a, s] * pw[a, s]/tot_pw_sex[s];

}

}

}

```

**fit**

```{r fit adjusted, echo=F, message=F, warning=F, include=TRUE, cache=T}

fit <- sampling(

mrp_adjusted,

thin = 1,

data = list(

y = y,

n = n,

pw = pw,

tot_pw_age = tot_pw_age,

tot_pw_sex = tot_pw_sex,

x = 166,

z = 901,

N_se = 179,

N_sp = 910),

chains = 3,

warmup = 1000,

iter = 10000,

cores = 3,

seed = 111,

pars = c("bage","bsex",

"sd_age",

"p_age","p_sex", "p_national"),

refresh = 0

)

options(scipen=999)

out_adj <- signif(summary(fit)$summary, 3)

out_adj <- out_adj[11:20, c(1, 4, 8)]

save.dta13(as.data.frame(out_adj), "out_adj.dta")
