# Supplementary material for: SARS-CoV-2 seroprevalence in three Kenyan health and demographic surveillance sites, December 2020-May 2021
Source: PLOS Glob Public Health. 2022 Aug 18;2(8):e0000883. doi: 10.1371/journal.pgph.0000883 (PMC10021917; doi:10.1371/journal.pgph.0000883)
Supplement: Attachment — Submitted filename: Reviewer comments.docx [file pgph.0000883.s010.docx]

PGPH-D-22-00247
SARS-CoV-2 Seroprevalence in three Kenyan Health and Demographic Surveillance Sites, December 2020-May 2021
PLOS Global Public Health

Dear Dr. Etyang,

Thank you for submitting your manuscript to PLOS Global Public Health. After careful consideration, we feel that it has merit but does not fully meet PLOS Global Public Health’s publication criteria as it currently stands. Therefore, we invite you to submit a revised version of the manuscript that addresses the points raised during the review process.

Please submit your revised manuscript by . If you will need more time than this to complete your revisions, please reply to this message or contact the journal office at [globalpubhealth@plos.org](mailto:globalpubhealth@plos.org). When you're ready to submit your revision, log on to <https://www.editorialmanager.com/pgph/> and select the 'Submissions Needing Revision' folder to locate your manuscript file.

Please include the following items when submitting your revised manuscript:

- A rebuttal letter that responds to each point raised by the editor and reviewer(s). You should upload this letter as a separate file labeled 'Response to Reviewers'.
- A marked-up copy of your manuscript that highlights changes made to the original version. You should upload this as a separate file labeled 'Revised Manuscript with Track Changes'.
- An unmarked version of your revised paper without tracked changes. You should upload this as a separate file labeled 'Manuscript'.

Guidelines for resubmitting your figure files are available below the reviewer comments at the end of this letter.

We look forward to receiving your revised manuscript.

Kind regards,

Hannah E. Clapham
Academic Editor
PLOS Global Public Health

Journal Requirements:

1. Your co-authors:
Ifedayo Adetifa  -[iadetifa@kemri-wellcome.org](mailto:iadetifa@kemri-wellcome.org)
Richard omore -[omorerichard@gmail.com](mailto:omorerichard@gmail.com)
Thomas Misore -[tmisore@kemricdc.org](mailto:tmisore@kemricdc.org)
Abdhalah Ziraba -[aziraba@aphrc.org](mailto:aziraba@aphrc.org)
Evelyn Gitau -[egitau@aphrc.org](mailto:egitau@aphrc.org)
John Gitonga -[jgitonga@kemri-wellcome.org](mailto:jgitonga@kemri-wellcome.org)
Bernadette Kutima -[BKutima@kemri-wellcome.org](mailto:BKutima@kemri-wellcome.org)
Henry Karanja -[HKaranja@kemri-wellcome.org](mailto:HKaranja@kemri-wellcome.org)
Monica Toroitich -[mtoroitich@kemri-wellcome.org](mailto:mtoroitich@kemri-wellcome.org)
James Tuju -[JTuju@kemri-wellcome.org](mailto:JTuju@kemri-wellcome.org)
Perpetual Wanjiku -[PWanjiku@kemri-wellcome.org](mailto:PWanjiku@kemri-wellcome.org)
Rashid Aman -[amanra54@gmail.com](mailto:amanra54@gmail.com)
Patrick Amoth -[patrickamoth@gmail.com](mailto:patrickamoth@gmail.com)
Mercy Mwangangi -[mukuimwangangi@gmail.com](mailto:mukuimwangangi@gmail.com)
Wangari Ng'ang'a -[nganga.wangari@gmail.com](mailto:nganga.wangari@gmail.com)
Charles Agoti -[CNyaigoti@kemri-wellcome.org](mailto:CNyaigoti@kemri-wellcome.org)
Lynette Ochola-Oyier -[LiOchola@kemri-wellcome.org](mailto:LiOchola@kemri-wellcome.org)
Amek Nyaguara -[ANyaguara@kemri-wellcome.org](mailto:ANyaguara@kemri-wellcome.org)
Sophie Uyoga -[SUyoga@kemri-wellcome.org](mailto:SUyoga@kemri-wellcome.org)
Katherine E Gallagher -[KGallagher@kemri-wellcome.org](mailto:KGallagher@kemri-wellcome.org)
Eunice W Kagucia -[ekagucia@kemri-wellcome.org](mailto:ekagucia@kemri-wellcome.org)
Benjamin Tsofa -[BTsofa@kemri-wellcome.org](mailto:BTsofa@kemri-wellcome.org)
Eric Maitha -[emaitha612@gmail.com](mailto:emaitha612@gmail.com)
Edwine Barasa -[EBarasa@kemri-wellcome.org](mailto:EBarasa@kemri-wellcome.org)
Philip Bejon -[PBejon@kemri-wellcome.org](mailto:PBejon@kemri-wellcome.org)
George M Warimwe -[GWarimwe@kemri-wellcome.org](mailto:GWarimwe@kemri-wellcome.org)
Ambrose Agweyu -[AAgweyu@kemri-wellcome.org](mailto:AAgweyu@kemri-wellcome.org)
,have not confirmed authorship of the manuscript. We have resent them the authorship confirmation email; however please check that the above email address for them is correct and follow up personally to ensure they confirm.

Please note that we cannot proceed your manuscript  until we have received confirmations from all co-authors.

This has been done.

**2. Please update the Funding Information tab in the system to matched with the Financial Disclosure.**

This has been done

**3. Please update the completed ‘Competing Interests’ statement, including any COIs declared by your co-authors. If you have no competing interests to declare, please state “The authors have declared that no competing interests exist”.**

This has been done.

**4. Please provide  separate figure files in .tif or .eps format only and remove any figures embedded in your manuscript file.  Please ensure that all files are under our size limit of 20MB.**

**For more information about how to convert your figure files please see our guidelines: Once you've converted your files to .tif or .eps, please also make sure that your figures meet our format requirements**

[**https://journals.plos.org/globalpublichealth/s/figures**](https://journals.plos.org/globalpublichealth/s/figures)

This has been done as per instructions.

**5. We notice that your supplementary [figures/tables] are included in the manuscript file.  Please remove them and upload them  with the file type 'Supporting Information'  . Please ensure that all Supporting Information files are included correctly and that each one has a legend listed in the manuscript after the references list.**

This has been done as per instructions.

**6. Please provide us with a direct link to the base layer of the map used in Fig S1 and ensure this location is also included in the figure legend.**

**Please note that, because all PLOS articles are published under a CC BY license (**[**creativecommons.org/licenses/by/4.0/**](http://creativecommons.org/licenses/by/4.0/)**), we cannot publish proprietary maps such as Google Maps, Mapquest or other copyrighted maps. If your map was obtained from a copyrighted source please amend the figure so that the base map used is from an openly available source.**

**Please note that only the following CC BY licences are compatible with PLOS licence: CC BY 4.0, CC BY 2.0  and CC BY 3.0, meanwhile such licences as CC BY-ND 3.0 and others are not compatible due to additional restrictions. If you are unsure whether you can use a map or not, please do reach out and we will be able to help you.**

**The following websites are good examples of where you can source open access or public domain maps:**

*** U.S. Geological Survey (USGS) - All maps are in the public domain. (**[**http://www.usgs.gov**](http://www.usgs.gov)**)**

*** PlaniGlobe - All maps are published under a Creative Commons license so please cite “PlaniGlobe,** [**http://www.planiglobe.com**](http://www.planiglobe.com)**, CC BY 2.0” in the image credit after the caption. (**[**http://www.planiglobe.com/?lang=enl**](http://www.planiglobe.com/?lang=enl)**)**

*** Natural Earth - All maps are public domain. (**[**http://www.naturalearthdata.com/about/terms-of-use/**](http://www.naturalearthdata.com/about/terms-of-use/)**)**

We used the Kenya boundaries outline shapefile from the Humanitarian data exchange platform [https://data.humdata.org/dataset/cod-ab-ken](https://eur01.safelinks.protection.outlook.com/?url=https%3A%2F%2Fdata.humdata.org%2Fdataset%2Fcod-ab-ken&data=05%7C01%7CAEtyang%40kemri-wellcome.org%7Cb7f1ffe9488f4025d2d008da22d5b855%7Ca5c0a820c8874727ac66403237d8c389%7C0%7C0%7C637860598553990472%7CUnknown%7CTWFpbGZsb3d8eyJWIjoiMC4wLjAwMDAiLCJQIjoiV2luMzIiLCJBTiI6Ik1haWwiLCJXVCI6Mn0%3D%7C3000%7C%7C%7C&sdata=NZEXF7fMtoMcLgJvS%2FtAZ64LV%2Fo1p%2FH6UmWgHOdVjBQ%3D&reserved=0)

Population data were downloaded in raster format from WorldPop at 1km spatial resolution [https://www.worldpop.org/](https://eur01.safelinks.protection.outlook.com/?url=https%3A%2F%2Fwww.worldpop.org%2F&data=05%7C01%7CAEtyang%40kemri-wellcome.org%7Cb7f1ffe9488f4025d2d008da22d5b855%7Ca5c0a820c8874727ac66403237d8c389%7C0%7C0%7C637860598553990472%7CUnknown%7CTWFpbGZsb3d8eyJWIjoiMC4wLjAwMDAiLCJQIjoiV2luMzIiLCJBTiI6Ik1haWwiLCJXVCI6Mn0%3D%7C3000%7C%7C%7C&sdata=u9%2BhCZZW8qJ9zZRHZ0dULK5lz0j7TFg%2FCRO30Tval7o%3D&reserved=0)

Additional Editor Comments (if provided):


[Note: HTML markup is below. Please do not edit.]

Reviewers' comments:

Reviewer's Responses to Questions

**Comments to the Author**

1. Does this manuscript meet PLOS Global Public Health’s [publication criteria](https://eur01.safelinks.protection.outlook.com/?url=https%3A%2F%2Fjournals.plos.org%2Fglobalpublichealth%2Fs%2Fcriteria-for-publication&data=04%7C01%7Caetyang%40kemri-wellcome.org%7C69431462841942b46a5408da216d42ff%7Ca5c0a820c8874727ac66403237d8c389%7C0%7C0%7C637859050409048083%7CUnknown%7CTWFpbGZsb3d8eyJWIjoiMC4wLjAwMDAiLCJQIjoiV2luMzIiLCJBTiI6Ik1haWwiLCJXVCI6Mn0%3D%7C3000&sdata=kgvVFtHHPUpTb6fWUPNC2WKh%2FDBTE%2B%2Bz7jAIh%2FzK0F4%3D&reserved=0)? Is the manuscript technically sound, and do the data support the conclusions? The manuscript must describe methodologically and ethically rigorous research with conclusions that are appropriately drawn based on the data presented.

Reviewer #1: Yes

Reviewer #2: Yes

2. Has the statistical analysis been performed appropriately and rigorously?

Reviewer #1: Yes

Reviewer #2: Yes

3. Have the authors made all data underlying the findings in their manuscript fully available (please refer to the Data Availability Statement at the start of the manuscript PDF file)?

The [PLOS Data policy](https://eur01.safelinks.protection.outlook.com/?url=https%3A%2F%2Fjournals.plos.org%2Fglobalpublichealth%2Fs%2Fdata-availability&data=04%7C01%7Caetyang%40kemri-wellcome.org%7C69431462841942b46a5408da216d42ff%7Ca5c0a820c8874727ac66403237d8c389%7C0%7C0%7C637859050409048083%7CUnknown%7CTWFpbGZsb3d8eyJWIjoiMC4wLjAwMDAiLCJQIjoiV2luMzIiLCJBTiI6Ik1haWwiLCJXVCI6Mn0%3D%7C3000&sdata=4i8KERIwJBUjyuOMjI71aCNIHjFtP9SHVoItKGk8Bqo%3D&reserved=0) requires authors to make all data underlying the findings described in their manuscript fully available without restriction, with rare exception. The data should be provided as part of the manuscript or its supporting information, or deposited to a public repository. For example, in addition to summary statistics, the data points behind means, medians and variance measures should be available. If there are restrictions on publicly sharing data—e.g. participant privacy or use of data from a third party—those must be specified.

Reviewer #1: No

Reviewer #2: No

4. Is the manuscript presented in an intelligible fashion and written in standard English?

PLOS Global Public Health does not copyedit accepted manuscripts, so the language in submitted articles must be clear, correct, and unambiguous. Any typographical or grammatical errors should be corrected at revision, so please note any specific errors here.

Reviewer #1: Yes

Reviewer #2: Yes

5. Review Comments to the Author

Please use the space provided to explain your answers to the questions above. You may also include additional comments for the author, including concerns about dual publication, research ethics, or publication ethics. (Please upload your review as an attachment if it exceeds 20,000 characters)

Reviewer #1: General comments:
This study provides useful data on SARS-CoV-2 seroprevalence from representative populations in Kenya, which adds to previous seroprevalence data from specific population subgroups (blood donors, truck drivers etc.) and includes data from both urban and rural settings. Available SARS-CoV-2 serological data are currently heavily skewed towards high-income settings and this study is a welcome addition to the literature. The analysis appears sound and the manuscript is for the most part clearly written. In some places the reader would benefit from greater detail, particularly in the methods, and the discussion section would be more compelling if the study findings were discussed in relation to the wider COVID-19 situation in the country.

**Major comments:**
**1.Introduction – it would help for context if the authors could provide some information on the circulating SARS-CoV-2 variants during the time of the study**
Thank you for this suggestion. The predominant variants at the time when the study was conducted were the alpha and beta variants. We have included this in the methods section (P7) and also added a supplementary figure (S2) displaying the timing of the different waves of infection in relation to when the study was conducted.

**2.Methods – If district-level data on daily/weekly case counts are available, I would suggest including a figure that shows this for each study site, with an indication on when the data collection period occurs. This would give some better contextual information about when in the epidemic samples were collected and how much transmission was occurring. Currently, I don’t find the information on median sample dates and reported cases particularly informative.**

Unfortunately, we do not have data on case counts at the level of spatial (district) and temporal(daily/weekly) granularity that the reviewer suggests. The data available are at County level, which we have reported in the methods. In addition the sites where the study was conducted do not fully cover the counties where the study was conducted. Supplementary Figure S2 which we have included in response to the first comment by the reviewer has national level case data which could help in placing the study in context.

3**.Methods, p6, para 2 – I feel the first sentence could be re-written slightly to make the target number of participants clearer without the reader having to do unnecessary mental arithmetic. Personally, I don’t find the sample size calculation particularly necessary or useful – the hypothesised seroprevalence is much lower than reality and doesn’t take into account uncertainties introduced by post-stratification weighting or adjustments for assay performance. It’s clear from the results that the expected estimates and margins of error are quite irrelevant, and I think the uncertainty bounds from the Bayesian analysis speak for themselves.**

We have revised the sentence to make it easier to understand. We acknowledge that the hypothesized seroprevalence turned out to be much lower than the actual seroprevalence when we conducted the study. However, at the time of designing the study we had little information to guide us in our sample size estimation. We believe that it is important to state our sample size assumptions in the interest of transparency.

**4.Methods, p8, para 1 – please clarify when assent was applicable, as well as the age of consent before which parental consent was required.**

This has been clarified as follows:

Written informed consent (and assent for individuals 13-17 years of age) was obtained from all participants, or their guardians (for individuals <18 years of age) before their participation in the study

**5.Methods – the statistical analyses section needs some more detail to make it clear what specific analyses were done. At the very least, the models used to estimate seroprevalence correcting for assay sensitivity and specificity should be detailed in the appendix, together with the assumed priors, simulation conditions, and what method was used to determine the point estimate and confidence bounds. There’s also mention of a logistic model for factors associated with seropositivity, but it’s not clear if this was a Bayesian model or not. The findings from this model also don’t seem to be presented in the results section. Could you also clarify what is meant by “weighted to account for underlying population, age, and sex structure”? Were factors other than age and sex accounted for in the weighting? If so, what were they?**

We have provided the Stan code that we used for the Bayesian model in the appendix, including the priors used. More details are available in the uploaded data and scripts provided with this revision for reproducibility purposes. Full details of the methods used to determine the point estimates and credible intervals, including weighting for population structure and assay performance are provided.

The logistic model to determine factors associated with seropositivity was a simple multivariable logistic model and not a Bayesian model. The results are presented in the supplementary appendix (Table S5).

**6.Results – could you please include the site-specific participation % in the first paragraph?**

This has been done.

**7.Table 1 – I feel this would be more compelling as a figure, perhaps showing the posterior estimates by time period as box/violin plots or something similar, and it would be easier to discern trends in relation to the uncertainty in the estimates. The table could be moved to the appendix.**

Thank you for this suggestion. We have produced a figure showing the trend in seroprevalence at the three sites over time (Figure 2). Table 1 has been moved to the appendix as Supplementary Table S1.

**8.Table 2 – in its present form, I don’t find this table to be very informative, because it’s clear that the survey wasn’t designed to provide reasonable estimates of seroprevalence within such fine age groupings. I also don’t think such fine age groupings are necessary, since epidemiologically, there’s not much reason to expect that 25-34 year-olds are very different to 35-44 year-olds in terms of SARS-CoV-2 infection risk. Children and the elderly are clearly different, but I would suggest combining adults into one or two categories. Presently, a number of these estimates are based on fewer than 10 individuals and the width of CrIs is larger than the point estimate in many cases. I would also be inclined to present this as a figure rather than a table (which again could be in the appendix), to allow for easier comparison across sites and age groups.**

Thank you for this suggestion. We have moved the table to the appendix (Supplementary Table S2). In its place we have included Figure 3 which displays the difference in seroprevalence between adults (≥16 years) versus children (< 16 years) at each of the three study sites for the entire study period.

**9.Results – in the methods, the authors say that information was collected on symptoms compatible with COVID-19. Was there any association between seropositivity and symptoms?**
We did not find any association between seropositivity and symptoms in a multivariate logistic model that adjusted for sex, age, location and education level. We have presented these results in supplementary table S5

**10.Discussion – it would be helpful to discuss the results in the context of the wider COVID-19 situation in the country, as well as comment on the possible reasons for differences in seroprevalence between sites. For example, how does the cumulative infection risk compare with the population risk of COVID-19 as determined by official statistics? And what are likely reasons why seroprevalence increased much more rapidly in Nairobi compared with the other two sites? It’s also interesting that seroprevalence in children was lower initially in Kilifi but rose dramatically over the study period. What might be the reasons for this? It’s not apparent in other age groups.**

Thank you for this suggestion. In response to this, we have added a paragraph in the discussion addressing these issues.

a
Minor comments:
1**.P8, para 2 – please clarify what platform (software, hardware) was used for electronic data collection**

We used android-run tablets to collect the data. Sentence has been modified to reflect this.

**2.P8, para 3 – “They” is ambiguous here, so suggest re-wording slightly.**

Corrected to read “study staff” instead of “they”

3**.P13, last para – antibody waning would lead to an underestimation of infection prevalence, rather than seroprevalence.**

Thank you for this, we have corrected the sentence so that it reads infection prevalence instead of seroprevalence.

Reviewer #2: This is a well-conducted and nicely written SARS-CoV-2 seroprevalence survey from Kenya, which uses robust sampling methodology. It provides useful insight into the extent of community spread of SARSCoV2 up to end of May 2021 and is a helpful contribution to the literature. I have minor suggestions only.

**1. Sampling: On page 7 it states potential participants that could not be found after 3 attempts were replaced by randomly selected individuals meeting same age and sex category as original individual. According to the Figure 1 flow chart (page 21) in Nairobi, of 2388 individuals approached, only 42% (981) were present at home. It would be helpful to know how many of the 2388 were from the original random selection. If less than 42% the sample becomes less random and more convenience based on who is at home. This has been addressed in the discussion, but it might be more transparent to add percentages in the 'Present at home' boxes of the flowchart.**
Thank you for this valid comment. In Nairobi we selected 3 random samples of 850 individuals so that we had one “original” sample list and two back up lists for individuals who could not be found from the first list. Approximately half of the 981 individuals that we found at home were from the original sample list while the other half were from the two back up lists. We have acknowledged this limitation in the discussion. We have included the percentages of those found at home in Figure 1 as suggested by the reviewer.

**2. Validation of the ELISA: the diagnostic test is well described and referenced but I found it difficult to establish what sort of patients sensitivity was evaluated on. Were the 174 SARSCoV2 PCR positive Kenyan adults all sick hospitalised patients? And any info re the panel from UK NIBSC? If they are broadly representative of recovered adults in the community then worth saying that. However if not, quoted assay sensitivity of 92.7% may be over-estimated (antibody titres will be higher if taken frm hospitalised patients soon after serious infection).**
Thank you for raising this important point. The validation was based on samples from 174 symptomatic PCR positive patients that were recovering from COVID-19. We have modified the methods section to make this point clearer. These are not necessarily broadly representative of individuals in the community. We had already acknowledged in the discussion the bias that this introduces in terms of underestimating infection prevalence (see 3^rd^ from last paragraph on page 15 of revised MS). We have also referenced an article (Bottomley et al, ref no. 27) in which we discuss this limitation in much more detail and introduces the concept of mixture modelling in an attempt to address the problem.

**3. Context: It would be helpful to get a bit more context of the epidemic in Kenya. E.g. Was it predominatly alpha variant circulating between December 2020 and May 2021? In the intro it mentions there were 5 waves up to Jan 22 but when did deaths peak according to official figures? In the intro it states only 5378 deaths have been reported. Given such high pre-vaccine seroprevalence figures this is indicative of either a very low case fatality rate or massive under-reporting of COVID-related deaths. The fact that 55.5% of >65s were antibody positive in Nairobi but relatively few individuals died of COVID warrants some discussion.**

Thank you. We have addressed these concerns in response to Reviewer #1’s comments, see above.

**4. The lower antibody prevalence in children also worth mentioning in the discussion. What do the authors think is reason for this? School closures? Or is it increased waning in this kids if they were the first to be affected in 2020? Is it possible that the lower blood volume in <5s (2ml vs 5ml) contributes to lower antibody positivity on the ELISA?**

Thank you for this comment. It is very unlikely that the lower blood volume drawn in children could have led to lower seropositivity in this group, as the amount used in the assay was 100µl for all individuals regardless of age. We think that the most likely explanation for the lower prevalence in children is because of age-related differences in immune response to infection with SARS-COV-2 which has been observed by other investigators. We have included a sentence in the discussion regarding this point.

6. PLOS authors have the option to publish the peer review history of their article ([what does this mean?](https://eur01.safelinks.protection.outlook.com/?url=https%3A%2F%2Fjournals.plos.org%2Fglobalpublichealth%2Fs%2Feditorial-and-peer-review-process%23loc-peer-review-history&data=04%7C01%7Caetyang%40kemri-wellcome.org%7C69431462841942b46a5408da216d42ff%7Ca5c0a820c8874727ac66403237d8c389%7C0%7C0%7C637859050409048083%7CUnknown%7CTWFpbGZsb3d8eyJWIjoiMC4wLjAwMDAiLCJQIjoiV2luMzIiLCJBTiI6Ik1haWwiLCJXVCI6Mn0%3D%7C3000&sdata=ZzEPQXv7HavzSLDzr5fFZr%2BpiEPlINXUnc7Ygmu5rJ4%3D&reserved=0)). If published, this will include your full peer review and any attached files.


**Do you want your identity to be public for this peer review?** If you choose “no”, your identity will remain anonymous but your review may still be made public.


For information about this choice, including consent withdrawal, please see our [Privacy Policy](https://eur01.safelinks.protection.outlook.com/?url=https%3A%2F%2Fwww.plos.org%2Fprivacy-policy&data=04%7C01%7Caetyang%40kemri-wellcome.org%7C69431462841942b46a5408da216d42ff%7Ca5c0a820c8874727ac66403237d8c389%7C0%7C0%7C637859050409048083%7CUnknown%7CTWFpbGZsb3d8eyJWIjoiMC4wLjAwMDAiLCJQIjoiV2luMzIiLCJBTiI6Ik1haWwiLCJXVCI6Mn0%3D%7C3000&sdata=rNoCk2a%2FNKoQuY%2BFsp8Ij4nUeJgePDd%2BSBv6p03BRlE%3D&reserved=0).

Reviewer #1: **Yes:** Clarence Tam

Reviewer #2: **Yes:** Barnaby Flower
